# Trajectories and timing of accelerated decline in specific memory domains preceding Alzheimer’s disease

**DOI:** 10.3389/fnagi.2026.1868433

**Published:** 2026-06-26

**Authors:** Xianxian Long, Meng Rong, Shushu Long, Shuqin Wen, Luhui Mo, Mingming Xu, Zeyun Zhang, Qinqi Nie

**Affiliations:** 1 Shenzhen Traditional Chinese Medicine Hospital, Shenzhen, China; 2 Guangzhou University of Chinese Medicine Fourth Clinical Medical College, Shenzhen, China; 3 Fuyang Center for Disease Control and Prevention, Fuyang, China; 4 Fujian Provincial Hospital, Fuzhou University Affiliated Provincial Hospital, Fuzhou, China; 5 Department of Scientific Research, Department of Neurology, Xiang’an Hospital of Xiamen University, School of Medicine, Xiamen University, Xiamen, China

**Keywords:** Alzheimer’s disease, generalized additive mixed models, influencing factors, longitudinal study, memory trajectory

## Abstract

**Introduction:**

Memory impairment is a hallmark feature of Alzheimer’s disease (AD) and may emerge years before clinical diagnosis. However, the temporal sequence and acceleration patterns of decline across specific memory domains during the preclinical stage remain incompletely understood. This study aimed to characterize the longitudinal dynamics of four distinct memory processes in individuals progressing to clinical AD, and to identify the temporal sequence of continually accelerated decline.

**Methods:**

We analyzed longitudinal data from 382 participants in the Alzheimer’s Disease Neuroimaging Initiative (ADNI) who converted from cognitively normal or mild cognitive impairment to AD. Generalized additive mixed models (GAMMs) were employed to characterize the nonlinear trajectories of four Rey Auditory Verbal Learning Test (RAVLT) derived scores over time prior to AD diagnosis, adjusting for key demographics and genetic factors. The finite difference method was applied to identify time points of continually accelerated decline from the fitted curves. Sensitivity analyses additionally adjusted for stroke and emotional incontinence, replicating all steps.

**Results:**

Our analysis revealed distinct nonlinear progression patterns across memory domains. All trajectories significantly deviated from linearity (Edf > 1, *p* < 0.001). Learning memory demonstrated the earliest continually accelerated decline, beginning 8.5 years before AD onset. APOE-ε4 allele carriage exhibited a dose-dependent effect, significantly associated with RAVLT_learning (β = −0.389 for one allele, −0.530 for two alleles) and RAVLT_forgetting (β = −0.449 for one allele, −0.508 for two alleles), but not with RAVLT_immediate performance. Females exhibited better immediate memory function than males, which was positively associated with years of education.

**Conclusion:**

The decline in four memory tests among individuals with preclinical AD followed nonlinear trajectories, with learning memory exhibiting the earliest continuously accelerated decline. Higher education was associated with better immediate memory performance, while the APOE-ε4 genotype specifically exacerbated impairments in learning memory and retention loss.

## 1 Introduction

Alzheimer’s disease (AD) is a neurodegenerative disorder primarily characterized by cognitive impairment and is the leading cause of dementia worldwide (Larson et al., 1992). According to the World Health Organization (WHO), more than 55 million people currently live with dementia worldwide, with a new case diagnosed approximately every 3 s (World Health Organization [WHO], 2026). The global number of individuals with dementia is projected to reach 139 million by 2050. AD is the most common form of dementia, accounting for approximately 60–70% of all cases and substantially exceeding the prevalence of other dementia subtypes (Akhter et al., 2021). Based on these estimates, approximately 33–38.5 million people currently living with AD worldwide, and this number is expected to increase to 83–97 million by 2050. These figures underscore the substantial global public health burden posed by AD. The increasing prevalence of AD highlights the urgent need for effective strategies for early detection and intervention to prevent disease onset or delay cognitive decline.

AD is characterized by a prolonged preclinical phase, during which pathological changes accumulate gradually and are accompanied by progressive cognitive decline (Bennett et al., 2002). As the disease progresses, multiple cognitive domains, including memory, language, and executive function, exhibit distinct patterns of deterioration (Bäckman et al., 2005; Williams et al., 2020). Memory impairment is the earliest and most prominent cognitive deficit associated with AD, often emerging years before clinical diagnosis (Cloutier et al., 2015). It is frequently observed as an early clinical manifestation and is considered an important predictor of the disease progression (Belleville et al., 2014; Peters et al., 2014). Memory can be characterized into several domains, including immediate recall, working memory, and long-term memory. Although most studies of memory trajectories have assumed linear patterns of decline, increasing evidence suggests that memory functions, particularly episodic memory, follow nonlinear trajectories before the clinical onset of AD, especially as individuals approach symptom onset (Smith et al., 2007). Smith et al. reported that episodic memory begins to decline approximately 6 years before AD onset, stabilizes until about 4 years before onset, and then undergoes accelerated decline beginning roughly 2 years before clinical manifestation (Grober et al., 2008; Smith et al., 2007). Although numerous studies have focused on compared the timing of accelerated decline across cognitive domains such as memory, executive function, and language (Amieva et al., 2005; Li et al., 2017; Yuan et al., 2024), few have examined the sequential order of sustained accelerated decline among specific memory subdomains. Characterizing the temporal trajectories and the sequence of sustained accelerated decline across memory subdomains before AD onset may improve understanding of cognitive changes during the preclinical stage and provide a foundation for cognitive monitoring and timely interventions in high-risk populations.

The etiology of AD remains incompletely understood. Although age, sex, educational attainment, and the APOE-ε4 allele have been established as important as important risk factors for AD (Adams et al., 2020; de Oliveira et al., 2014; Sando et al., 2008), their specific effects on different memory domains remain unclear. Understanding how these factors influence the nonlinear decline trajectories of memory subdomains in individuals who eventually convert to AD is essential for developing longitudinal models that accurately characterize disease progression in this population and support personalized diagnostic and therapeutic strategies. Therefore, this study aimed to characterize the trajectories and key sustained transition points of different memory subdomains before the onset of AD and to determine the effects of age, sex, educational attainment, and the APOE-ε4 status on these trajectories.

## 2 Participants and methods

### 2.1 Participants

Data were acquired through the Alzheimer’s Disease Neuroimaging Initiative (ADNI) database with follow-up from 2005 through 2024.^1^ The database was established to develop standardized clinical, neuroimaging, genetic, and biochemical biomarkers for early AD detection and progression monitoring. It includes cognitively normal (CN), mild cognitive impairment (MCI), and AD individuals aged 55–90 years (Weiner et al., 2013). The baseline MCI participants undergo follow-up assessments every 6 months for the first 2 years, followed by annual evaluations thereafter. In contrast, CN participants are monitored biannually during the initial year and transitioned to annual assessments starting from year 2.^2^

**FIGURE 1:**
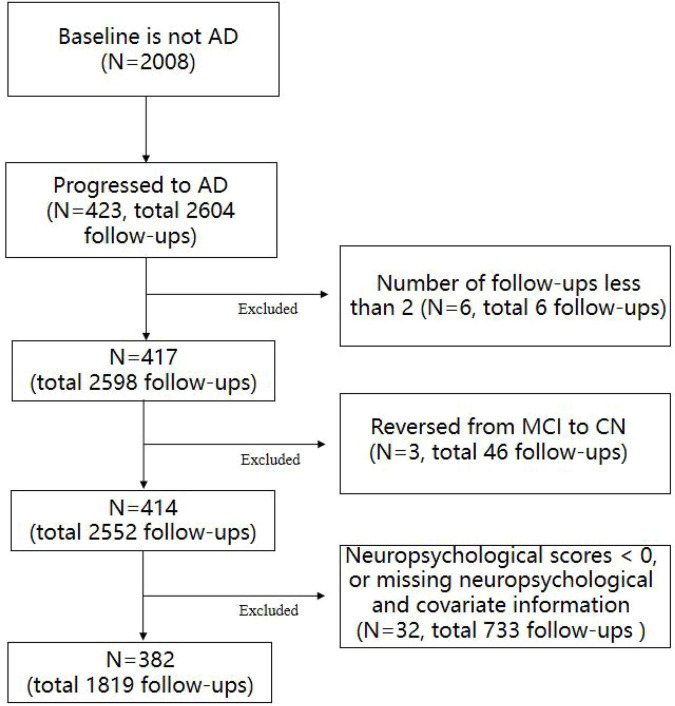
Workflow of study subject enrollment.

Participants were diagnosed with CN or MCI at baseline, and those who progressed to AD during follow-up were included in the study. We excluded patients as follows: (1) fewer than two follow-up visits, (2) reversion from MCI to CN status, (3) neuropsychological scores <0, or missing neuropsychological and covariate information. Through this standardized selection protocol, 382 eligible participants were ultimately included in the analysis. The detailed enrollment workflow is illustrated in Figure 1.

### 2.2 Cognitive performance

RAVLT is a list-learning task designed to measure auditory verbal memory, employing two 15-item unrelated word lists (List A and List B) administered to participants over multiple trials (Gonzalez et al., 2021). Immediate memory is measured through the total scores of trials 1–5 from List A (RAVLT_immediate). The total score ranges from 0 to 75, with lower scores indicating worse immediate memory performance (Reitan, 1958). Learning memory is measured by the difference between trial 5 score and trial 1 score from List A (RAVLT_learning), while retention loss is measured by the difference between trial 5 score and the 30-min delayed recall score (RAVLT_forgetting). Both metrics independently range from 0 to 15, with higher learning memory scores indicating better cognitive performance and higher retention loss scores reflecting worse cognitive performance. Rate of retention loss (RAVLT_perc_forgetting) is calculated as the ratio of the RAVLT_forgetting score to the trial 5 score from List A. To unify the interpretation direction of cognitive scores, we reversed the original RAVLT_forgetting and RAVLT_perc_forgetting scores by considering their negative values, so that higher reversed scores represent better cognitive function.

### 2.3 Covariates

Baseline age, education, body mass index (BMI), race (white/other), marital status (married/other), number of APOE-ε4 allele (0–2), history of hypertension (yes/no), and retirement (yes/no) were adjusted for in the primary models. History of stroke (yes/no) and emotional incontinence (yes/no) were adjusted for in sensitivity analyses.

### 2.4 Time of sustained acceleration

The time of sustained acceleration (turning point) was defined as the point at which the second derivative equaled zero and both the first and second derivatives remained ≤ 0 thereafter.

### 2.5 Statistical analysis

Continuous variables are summarized as mean ± standard deviation (SD) with corresponding range, while categorical variables are expressed as frequencies and percentages. Generalized additive mixed models (GAMM) were employed to analyze influencing factors affecting scores of four memory-related cognitive functions, and to describe the trajectory of these neuropsychological scores prior to AD onset. GAMM, as a hybrid statistical framework integrating generalized additive models with linear mixed-effects analysis, offers methodological flexibility by relaxing parametric assumptions (Herting et al., 2018; Yang et al., 2012). Specifically, GAMM was fitted using “gamm()” function from the “mgcv” package. The smooth term for time prior to AD used a cubic regression spline, with smoothing parameters selected automatically via restricted maximum likelihood. The model included a random intercept per participant, and no random slopes were added. Time prior to AD onset was entered in its original year metric, without centering or scaling. Only main effects were modeled, and interactions with time were not tested. Missing data were handled using listwise deletion.

To avoid uncertainties from integrated functions, a stepwise strategy was used to estimate derivatives and their confidence intervals. First, the “derivatives()” function (forward difference) from “gratia” package generated 200 evenly spaced points across follow-up time, approximating the first derivative via difference quotients. To control for multiple comparisons, we assumed model coefficients followed a multivariate normal distribution and performed 10,000 parametric simulations to construct 95% simultaneous confidence bands. Second, the second derivative was manually calculated via forward difference, with its confidence intervals approximated via error propagation. Finally, to reduce numerical noise, a penalized cubic regression spline was fitted to the second-derivative curve, and “uniroot.all()” located its zeros and turning point. Using the identified turning point as the center, a ± 1.5-year local window (matching the follow-up interval) was defined. Within this window, the continuous region where the point-wise 95% confidence band of the second derivative contained zero was identified as the 95% confidence interval for the turning point. In sensitivity analyses, we additionally adjusted for history of stroke and emotional incontinence and replicated all the above analyses. All statistical analyses were performed using R-4.2.1, and a *p* < 0.05 was considered statistically significant.

## 3 Results

### 3.1 Participant characteristics

The study cohort comprised 382 participants who transitioned from CN or MCI to AD during the follow-up period. They had a mean visitation number of 4.76 ± 2.46 times (range 2.00–14.00), with a total of 1,819 visits. The baseline mean age was 73.97 ± 6.90 years (range 55.00–88.40), while AD onset occurred at a mean age of 78.50 ± 7.50 years (range 56.00–94.70). The sample was predominantly male (60.73%), white (95.29%), and married (81.41%), with a mean educational attainment of 15.92 ± 2.76 years (range 6.00–20.00). In addition, genetic analysis revealed that 239 (62.57%) of all participants carried at least one APOE-ε4 allele. Baseline neuropsychological measure scores on the RAVLT subdomains demonstrated mean scores of 30.77 for RAVLT_immediate, 3.49 for RAVLT_learning, −5.05 for RAVLT_forgetting, and for −72.12% RAVLT_perc_forgetting (Table 1).

**TABLE 1:** Basic information of the 382 participants.

Variables^a^	M (SD)/N (%)^d^	Range
Number of visits	4.76 (2.46)	2.00–14.00
Length of follow-up (year)	2.48 (2.57)	0.50–13.00
Age at AD onset (year)	78.50 (7.50)	56.00–94.70
RAVLT_immediate	30.77 (9.01)	11.00–67.00
RAVLT_learning	3.49 (2.41)	0–11.00
RAVLT_forgetting	−5.05 (2.22)	−13.00 to 0
RAVLT_perc_forgetting (%)	−72.12 (29.31)	−100.00 to 0
Education (year)	15.92 (2.76)	6.00–20.00
Baseline age (year)	73.97 (6.90)	55.00–88.40
BMI	19.93 (4.83)	12.40–43.96
Gender		
Male	232 (60.73)	
Female	150 (39.27)	
Race		
White	364 (95.29)	
Others^b^	18 (4.71)	
Marital status		
Married	311 (81.41)	
Others^c^	71 (18.59)	
Number of APOE-ε4		
0	143 (37.43)	
1	184 (48.17)	
2	55 (14.40)	
History of hypertension		
Yes	188 (49.21)	
No	194 (50.79)	
Retirement		
Yes	321 (84.03)	
No	61 (15.97)	

^a^Data for baseline age, BMI, RAVLT_immediate, RAVLT_learning, RAVLT_forgetting, RAVLT_perc_forgetting, education, race, marital status, history of hypertension and retirement are baseline information.

^b^Includes black people, Asians, and mixed-race individuals.

^c^Includes divorce, widowhood and unmarried.

^d^Mean (SD) for continuous variables and *n* (%) for discrete variables.

**FIGURE 2:**
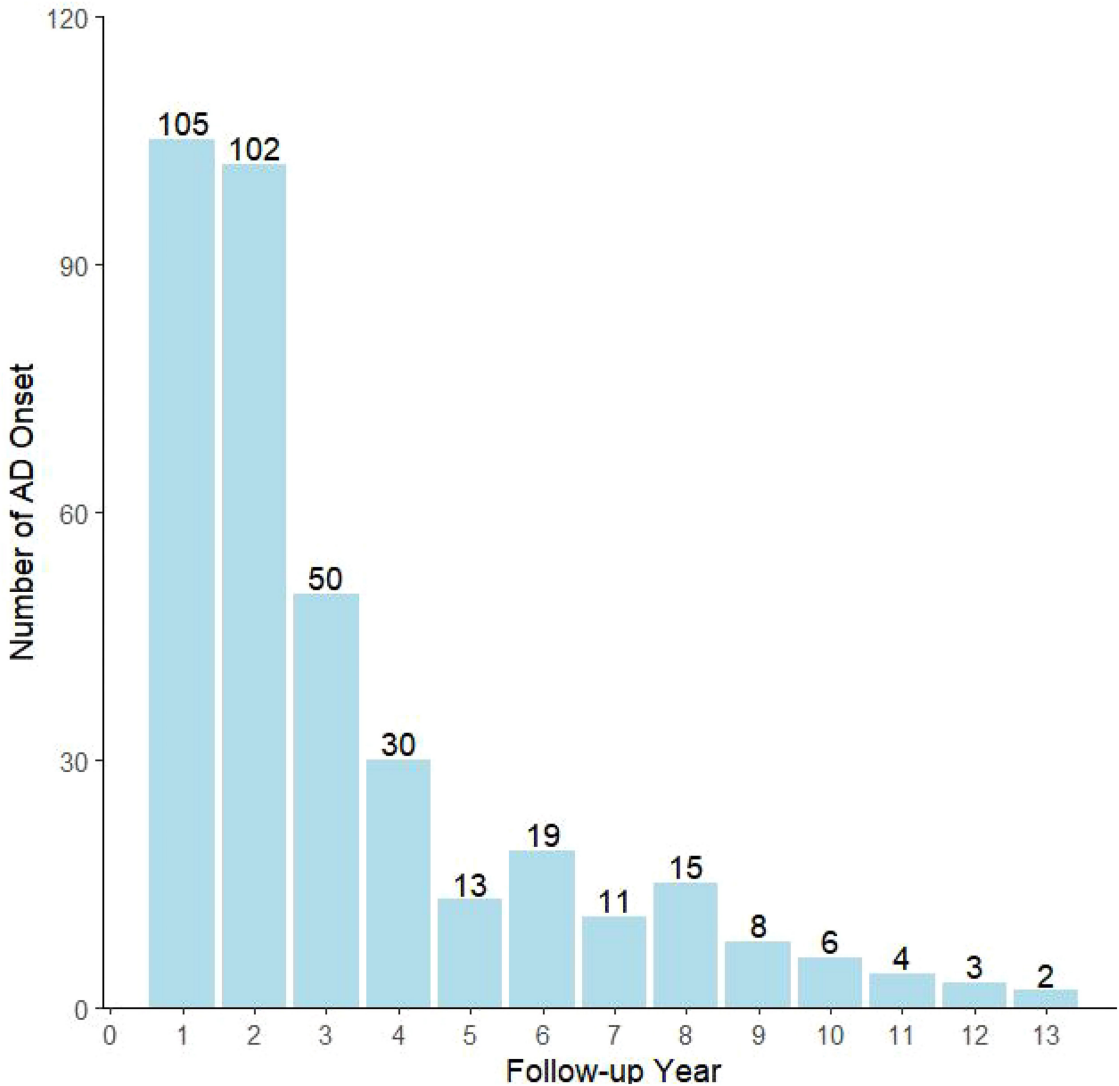
Number of AD onset participants at follow-ups.

Figure 2 showed the number of AD onset participants at follow-up. The shortest time to progression to AD was 0.50 years, while the longest was 13.00 years during follow-up. Most participants (67.28%) progressed to AD within the first 3 years. The mean time to AD progression was 2.48 ± 2.57 years (median 2.00).

### 3.2 GAMM analysis of changes in cognitive function scores

Table 2 summarizes the key findings from the GAMM analyzing four memory-related cognitive functions. The results revealed significant gender differences in RAVLT_immediate performance, with men exhibiting lower scores than women (β = −2.733, *p* < 0.001). Educational year showed a robust positive association with RAVLT_immediate scores (β = 0.440, *p* < 0.001), indicating that each additional year of education was linked to an approximate 0.440-point increase in immediate memory.

**TABLE 2:** The results of GAMM model analyses with four neuropsychological measures.

Covariates	RAVLT_immediate		RAVLT_learning		RAVLT_forgetting		RAVLT_perc_forgetting	
	β	*p*	β	*p*	β	*p*	β	*p*
Intercept	29.649	< 0.001	2.523	0.046	−5.984	< 0.001	−84.799	< 0.001
Age	−0.059	0.272	0.001	0.940	0.017	0.140	0.066	0.680
Gender (ref = Female)								
Male	−2.733	< 0.001	−0.328	0.075	0.145	0.380	−1.416	0.539
Educational year	0.440	< 0.001	0.053	0.080	−0.043	0.113	0.202	0.591
Race (ref = Othersa)								
White	−0.235	0.884	0.290	0.450	−0.112	0.746	−2.459	0.611
Marital status (ref = Othersb)								
Married	0.646	0.493	0.179	0.425	0.317	0.117	4.098	0.147
APOE-ε4 (ref = 0)								
1	−1.17	0.131	−0.389	0.028	−0.449	0.005	−7.958	0.004
2	−1.573	0.141	−0.530	0.039	−0.508	0.028	−10.019	0.002
History of hypertension (ref = No)								
Yes	−0.806	0.253	−0.256	0.128	0.003	0.983	−0.986	0.641
Retirement (ref = No)								
Yes	0.392	0.682	−0.089	0.696	−0.222	0.279	−1.852	0.518
BMI	−0.046	0.525	0.001	0.957	0.030	0.061	0.358	0.103
**Spline fit**	***Edf***	***p***	***Edf***	***p***	***Edf***	***p***	***Edf***	***p***
*S* (Time prior to AD)	4.966	< 0.001	2.095	< 0.001	3.086	< 0.001	3.109	< 0.001

“*S* ()” refers to the smoothing function from the generalized additive mixed models, “*Edf*” estimated effective degrees of freedom, *p* < 0.05 is significant. Data for RAVLT_immediate, RAVLT_learning, RAVLT_forgetting, and RAVLT_perc_forgetting are all longitudinal data. Data for age, education, race, marital status, history of hypertension, retirement and BMI are baseline information.

*^a^*Includes black people, Asians, and mixed-race individuals,

^b^ includes divorce, widowhood and unmarried.

Furthermore, both 1 and 2 APOE-ε4 allele carriers showed lower scores on RAVLT_learning, RAVLT_forgetting and RAVLT_perc_forgetting than non-carriers. Specifically, compared to non-carriers, individuals carrying one APOE-ε4 allele had lower scores on RAVLT_learning (by 0.389), RAVLT_forgetting (by 0.449), and RAVLT_perc_forgetting (by 7.958); those carrying two APOE-ε4 alleles showed even greater reductions: lower scores on RAVLT_learning (by 0.530), RAVLT_forgetting (by 0.508), and RAVLT_perc_forgetting (by 10.019). These results indicate that the more APOE-ε4 alleles an individual carries, the more impairments they experience in learning and retention loss, alongside accelerated rate of retention loss. Notably, the associations between time prior to AD onset and performance on four tests exhibited significant nonlinearity (*Edf* > 1, *p* < 0.05 for all models), suggesting that four memory-related cognitive decline trajectories deviated from linear patterns during the preclinical AD phase.

### 3.3 Trajectories of neuropsychological measure scores

**FIGURE 3:**
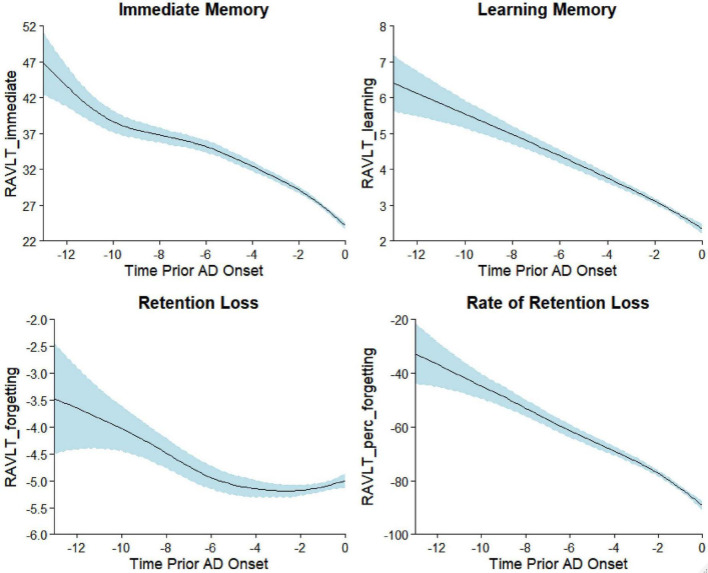
Trajectories of four neuropsychological measure scores prior to AD onset based on the result of GAMM. The black curves represent the fitted values of GAMM, the blue band denotes the 95% confidence interval.

Figure 3 visualizes the predicted trajectories of the four memory-related neuropsychological measure scores prior to AD onset, all of which exhibited nonlinear patterns over time.. Overall, the trajectory scores of all four memory-related cognitive functions declined over time, reflecting an overall impairment of these functions during the disease progression (Figure 3). Specifically, the decline of the RAVLT_immediate score trajectory initially accelerated, then decelerated, before accelerating again; the RAVLT_learning and RAVLT_perc_forgetting trajectory exhibited a slight accelerated decline; the RAVLT_forgetting trajectory first accelerated decline, then decelerated, and finally the scores begin to increase (Figure 3 and Supplementary Figure 2). Additionally, to aid reader comprehension, we also plotted the participant-level raw longitudinal trajectories of the four neuropsychological scores prior to AD onset in Supplementary Figure 1.

**FIGURE 4:**
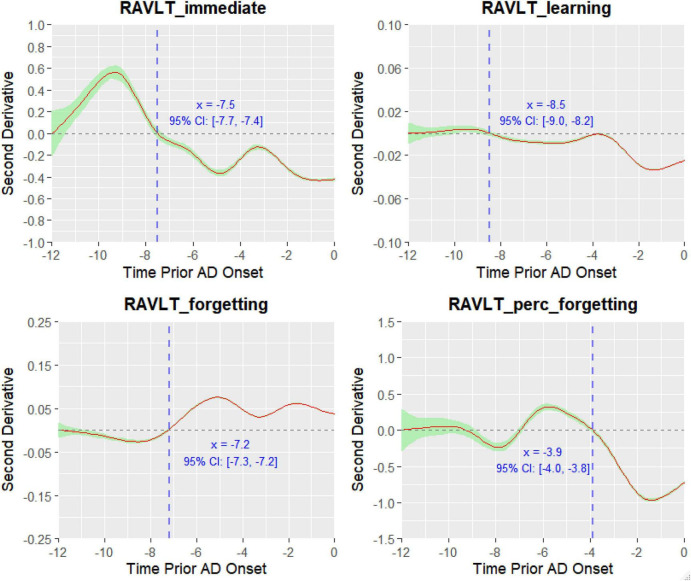
The second-order derivative values of GAMM-fitted trajectories for four neuropsychological scores prior to AD onset. The solid red line represents the second derivative curve of the fitted values of the GAMM. The green band denotes the 95% confidence interval for the second derivative. The blue dashed line is the vertical line drawn at the *x*-axis location of the turning point of sustained accelerated decline. The blue text indicates the 95% confidence interval for the turning point on the *x*-axis.

Figure 4 illustrates the second-order derivative values and their confidence intervals of GAMM-fitted trajectories for four neuropsychological scores prior to AD onse, as well as the confidence intervals for the inflection points on the *x*-axis. The second derivative of RAVLT_immediate and RAVLT_learning scores decreased to zero at 7.5 (95%CI: −7.7, −7.4) and 8.5 (95%CI: −9.0, −8.2) years before onset, respectively, and became negative thereafter. The velocity of their score trajectories at these time points shifted toward more negative values, indicating that these are the time points when their trajectories began to exhibit progressively accelerating decline. For the RAVLT_forgetting score, the second derivative increased to zero at 7.2 years before onset (95%CI: –7.3, –7.2) and became positive thereafter. Combined with its first derivative gradually increasing from negative to positive, this indicates that retention loss began to exhibit a sustained deceleration of decline starting from 7.2 years before onset (Supplementary Figure 2). For the RAVLT_perc_forgetting score, the second derivative showed multiple fluctuations and began to gradually decrease after 3.9 years before onset (95%CI: –4.0, –3.8), indicating that the rate of retention loss started to show a sustained accelerated decline from 3.9 years before onset.

In sensitivity analyses, we additionally adjusted for history of stroke and emotional incontinence and repeated the above analyses. The results showed similar outcomes in Table 2 and Supplementary Table 1, as well as comparable trajectory patterns in Figure 3 and Supplementary Figure 3, and in Figure 4 and Supplementary Figure 5. This consistency indicates that our findings are robust and that additional covariate adjustments had little impact on our results.

## 4 Discussion

We employed GAMM to systematically track the longitudinal trajectories of four memory-related cognitive functions—immediate memory, learning memory, retention loss, and rate of retention loss—in individuals who progressed to AD during follow-up. Our analysis not only confirmed the nonlinear nature of cognitive decline but, more importantly, revealed heterogeneous decline patterns and critical turning points across different memory dimensions during the preclinical phase. These findings offer new perspectives for understanding the cognitive evolution of preclinical AD.

A key finding to be emphasized is the nonlinear nature and dimensional heterogeneity of memory function decline. The trajectories of all four memory-related scores over time significantly deviated from linearity (all nonlinear terms *Edf* > 1, *p* < 0.001), characterized by a period of stability or subtle decline over several years, followed by accelerated deterioration approaching the time of diagnosis, similar to previous findings (Cloutier et al., 2015; Yuan et al., 2024). More importantly, the trajectories of the four memory-related scores over time differed. The decline in RAVLT_immediate followed a pattern of acceleration, then deceleration, followed by accelerated decline again. RAVLT_learning and RAVLT_perc_forgetting exhibited a pattern of continuous, mild accelerated decline. RAVLT_forgetting displayed an initial accelerated decline, then deceleration, and finally an increase in scores. These findings suggest that assessment based on a single dimension may be insufficient to fully capture the characteristics of memory change. Future research could build upon the approach of Ren et al. (2025) by integrating multimodal imaging and biomarkers to construct a comprehensive predictive framework that incorporates multiple memory dimensions, thereby providing a basis for early identification and precise intervention in AD.

In terms of the timing of decline, learning memory was the earliest to exhibit continuously accelerated decline (8.5 years before onset), whereas rate of retention loss was the latest to emerge (4.3 years before onset). Compared with previous research, the time points of accelerated decline identified in our study show slight discrepancies (Grober et al., 2019). Williams et al. used linear and nonlinear mixed models to analyze 165 AD cases from the Baltimore Longitudinal Study of Aging (BLSA) and found that the first inflection point for immediate memory occurred earliest, at 11.65 years before onset, while the first inflection point for delayed memory occurred at 7.58 years before onset (Williams et al., 2020). Grober et al. (2019) employed Change-Point Analysis to examine 217 AD cases from the BLSA and found that the first inflection point for delayed free recall occurred earliest, at 7.29 years before diagnosis, followed by learning at 6.58 years, and rate of retention loss at 5.3 years before diagnosis (Grober et al., 2019). These discrepancies may stem from differences in measurement tools and sample composition. While we used RAVLT-derived scores, Grober et al. employed a different memory test, and although Williams et al. also utilized RAVLT, their metric composition varied. Additionally, our findings are based on the ADNI cohort, whereas Williams and Grober et al. analyzed BLSA data. Kang et al. reported that cohort heterogeneity (e.g., enrollment criteria, follow-up intervals, age distribution) results in varying temporal sequences of cognitive decline across single-cohort studies (Kang et al., 2025). Additionally, this study employed second-order derivatives to identify sustained directional changes in trajectories, estimating the periods of continuously accelerated or decelerated decline across multiple memory functions, rather than simply identifying inflection points of rate of change. This distinction in the direction of sustained change offers a novel perspective for understanding the dynamic evolution of cognitive reserve: early compensation may delay the onset of decline in specific domains, yet it cannot alter the overall trajectory of eventual accelerated deterioration.

The association between the APOE-ε4 allele and cognitive function may exhibit domain-specificity and a dose-dependent relationship. Certain cognitive domains may be more predisposed to be influenced by APOE genotype than others (Small et al., 2004). The cross-sectional study by Todd et al. (2018) employing domain-specific cognitive assessments revealed that APOE-ε4 genotype may be more closely associated with working memory and declarative learning than with attention. Our findings demonstrated that the APOE-ε4 allele was significantly associated with impairments in learning memory and retention loss and a higher rate of retention loss, but not with immediate memory, highlighting the selective effect of this genotype on cognition. A dose-dependent pattern was also evident, with increasing allele count linked to greater deficits in these domains. Greenwood et al. reported similar findings, demonstrating that performance on a visuospatial attention task exhibited a graded decline with an increasing number of APOE-ε4 alleles, whereas working memory was significantly impaired only in ε4 homozygotes (Greenwood et al., 2005). Recent evidence from the ADNI cohort further demonstrated a dose-dependent effect of APOE-ε4 on episodic memory decline—homozygotes showed steeper decline than heterozygotes—whereas no such pattern emerged for processing speed, executive function, or language (Vanderlip and Stark, 2024). Of note, immediate memory did not exhibit a significant dose-dependent effect in our study, further strengthening the evidence for domain specificity.

Equally important, sex and education emerged as significant predictors of immediate memory performance. Our finding that females performed better than males on the RAVLT_immediate memory test is consistent with prior literature (Aartsen et al., 2004; Ratigan et al., 2016; Sundermann et al., 2016). Belachew et al. (2025) reported a sex-specific effect in the hippocampus-memory link: the correlation was significantly stronger in females, and at any given hippocampal volume, women showed superior memory performance compared to men (Belachew et al., 2025). Females had larger hippocampal volumes relative to males, while males demonstrated larger volumes in the amygdala and thalamus (Neufang et al., 2009). Furthermore, substantial preclinical and clinical evidence suggests that neuroactive sex steroid hormones, including 17β-estradiol, play a role in learning and memory in females and may underlie sex differences in performance (Brinton, 2009; Liu et al., 2008). The female hippocampus is characterized by both larger volume and a greater concentration of estrogen receptors compared to males (Neufang et al., 2009). Estrogen influences memory function by affecting brain structures throughout memory circuits, such as increasing cerebral blood flow, promoting neuronal growth and axon regeneration, and modulating amyloid accumulation (Jacobs et al., 2017; Shanmugan and Epperson, 2014). Sex differences in memory function, particularly in verbal memory, emerge in childhood, magnify after puberty, persist into adulthood, and decline following menopause, though a modest female advantage remains (Bleecker et al., 1988; Kramer et al., 1997; van Hooren et al., 2007). In early middle age, women outperformed age-matched men on certain memory tests, but this advantage diminished after menopause (Bramen et al., 2011; Li and Singh, 2014; Rentz et al., 2017). Variations in memory function parallel physiological changes in estrogen levels, further suggesting that estrogen may play a significant role in sex differences in memory.

Regarding the relationship with education, higher educational attainment was associated with better immediate recall performance, a finding consistent with previous studies (Yuan et al., 2024). Our results support the cognitive reserve hypothesis, wherein more years of education may confer resistance to early memory decline. According to this theory, factors like higher IQ, education, socioeconomic status, and occupational achievement help buffer against neuropathology by engaging alternative neural networks or cognitive strategies (Stern et al., 1994; Stern et al., 2003). According to this framework, cognitive reserve serves as a buffer against neuropathological damage in highly educated individuals, delaying symptom onset. However, this does not imply that the brain is immune to injury. When the pathological burden exceeds the brain’s compensatory capacity, cognitive function still declines sharply, particularly among women with lower educational levels (Rodríguez-Camacho et al., 2025).

Although our study observed clear nonlinear trajectories and temporal differences, providing new insights into the sequence of cognitive changes in preclinical AD, some limitations remain due to the study design and sample characteristics. First, the statistical framework of this study required aligning participants’ longitudinal trajectories based on the time of their AD diagnosis. However, the minimum follow-up interval of 6 months in the ADNI data may result in imprecise localization of AD onset, potentially introducing bias into the results. Second, the study sample exhibited relative homogeneity in demographic characteristics, with participants predominantly consisting of white individuals and those with higher education levels, which may limit the generalizability of the findings. Therefore, our conclusions are primarily applicable to ADNI-like populations. Nevertheless, these results may provide a theoretical basis for future investigations into different memory subdomains in Asian populations with cognitive impairment. Third, we did not account for factors such as psychiatric conditions, chronic diseases, or the number of comorbidities. Depression, chronic diseases, and the number of comorbidities are closely associated with cognitive decline (Ma et al., 2024; Wang et al., 2025). These unaccounted confounding factors may influence the trajectories of memory decline. Future studies should validate this temporal model in multicenter cohorts with greater demographic diversity and integrate it with cross-modal neuroimaging markers, including cerebrospinal fluid biomarkers, amyloid-PET, and tau-PET. The goal is to develop a multidimensional and comprehensive early warning system capable of dynamically predicting the risk and timeline of AD conversion.

This study reveals distinct nonlinear trajectories and temporal sequences of memory decline in preclinical AD, with learning ability emerging as the earliest cognitive sentinel exhibiting continuously accelerated decline. Higher education was associated with better immediate memory performance, while the APOE-ε4 genotype specifically exacerbates impairments in learning memory and retention loss. In individuals who eventually convert to AD, these findings provide a refined, domain-specific timeline of cognitive decline in preclinical AD, offering valuable insights for developing early intervention strategies.

## Data Availability

The datasets presented in this study can be found in online repositories. The names of the repository/repositories and accession number(s) can be found at: For the updated information on ADNI, please visit the official website: https://adni.loni.usc.edu/about/.

## Footnotes

1
https://adni.loni.usc.edu/

2
https://adni.loni.usc.edu/wp-content/uploads/2010/09/ADNI_GeneralProceduresManual.pdf

## References

[B1] Aartsen M. J. Martin M. Zimprich D. (2004). Gender differences in level and change in cognitive functioning. Results from the longitudinal aging study Amsterdam. *Gerontology* 50 35–38. 10.1159/000074387 14654725

[B2] Adams L. D. Slifer S. H. Ramos J. Inciute J. D. Starks T. D. Scott A. M. et al. (2020). Education and its effect on risk and age at onset in Alzheimer disease (AD) in African Americans. *Alzheimers Dement.* 16:e046078. 10.1002/alz.046078

[B3] Akhter F. Persaud A. Zaokari Y. Zhao Z. Zhu D. (2021). Vascular dementia and underlying sex differences. *Front. Aging Neurosci.* 13:720715. 10.3389/fnagi.2021.720715 34566624 PMC8457333

[B4] Amieva H. Jacqmin-Gadda H. Orgogozo J. M. Le Carret N. Helmer C. Letenneur L. et al. (2005). The 9 year cognitive decline before dementia of the Alzheimer type: A prospective population-based study. *Brain* 128 1093–1101. 10.1093/brain/awh451 15774508

[B5] Bäckman L. Jones S. Berger A. K. Laukka E. J. Small B. J. (2005). Cognitive impairment in preclinical Alzheimer’s disease: A meta-analysis. *Neuropsychology* 19 520–531. 10.1037/0894-4105.19.4.520 16060827

[B6] Belachew A. Biber S. Kukull W. W. Mukherjee S. Crane P. K. Mez J. et al. (2025). Sex and APOE genotype differentially modify the association between hippocampal volume and memory performance. *Alzheimers Dement.* 21:e104990. 10.1002/alz70855_104990

[B7] Belleville S. Gauthier S. Lepage E. Kergoat M. J. Gilbert B. (2014). Predicting decline in mild cognitive impairment: A prospective cognitive study. *Neuropsychology* 28 643–652. 10.1037/neu0000063 24588699

[B8] Bennett D. A. Wilson R. S. Schneider J. A. Evans D. A. Beckett L. A. Aggarwal N. T. et al. (2002). Natural history of mild cognitive impairment in older persons. *Neurology* 59 198–205. 10.1212/wnl.59.2.198 12136057

[B9] Bleecker M. L. Bolla-Wilson K. Agnew J. Meyers D. A. (1988). Age-related sex differences in verbal memory. *J. Clin. Psychol.* 44 403–411. 10.1002/1097-4679(198805)44:3<403::aid-jclp2270440315>3.0.co;2-0 3384968

[B10] Bramen J. E. Hranilovich J. A. Dahl R. E. Forbes E. E. Chen J. Toga A. W. et al. (2011). Puberty influences medial temporal lobe and cortical gray matter maturation differently in boys than girls matched for sexual maturity. *Cereb. Cortex* 21 636–646. 10.1093/cercor/bhq137 20713504 PMC3041011

[B11] Brinton R. D. (2009). Estrogen-induced plasticity from cells to circuits: Predictions for cognitive function. *Trends Pharmacol. Sci.* 30 212–222. 10.1016/j.tips.2008.12.006 19299024 PMC3167490

[B12] Cloutier S. Chertkow H. Kergoat M. J. Gauthier S. Belleville S. (2015). Patterns of cognitive decline prior to dementia in persons with mild cognitive impairment. *J. Alzheimers Dis.* 47 901–913. 10.3233/JAD-142910 26401770 PMC4923749

[B13] de Oliveira F. F. Bertolucci P. H. Chen E. S. Smith M. C. (2014). Risk factors for age at onset of dementia due to Alzheimer’s disease in a sample of patients with low mean schooling from São Paulo, Brazil. *Int. J. Geriatr. Psychiatry* 29 1033–1039. 10.1002/gps.4094 24596166

[B14] Gonzalez C. Tommasi N. S. Briggs D. Properzi M. J. Amariglio R. E. Marshall G. A. (2021). Financial capacity and regional cerebral tau in cognitively normal older adults, mild cognitive impairment, and Alzheimer’s disease dementia. *J. Alzheimers Dis.* 79 1133–1142. 10.3233/JAD-201122 33386806 PMC7870560

[B15] Greenwood P. M. Lambert C. Sunderland T. Parasuraman R. (2005). Effects of apolipoprotein E genotype on spatial attention, working memory, and their interaction in healthy, middle-aged adults: Results from the national institute of mental health’s BIOCARD study. *Neuropsychology* 19 199–211. 10.1037/0894-4105.19.2.199 15769204 PMC1350931

[B16] Grober E. An Y. Lipton R. B. Kawas C. Resnick S. M. (2019). Timing of onset and rate of decline in learning and retention in the pre-dementia phase of Alzheimer’s disease. *J. Int. Neuropsychol. Soc.* 25 699–705. 10.1017/S1355617719000304 31094304 PMC6747692

[B17] Grober E. Hall C. B. Lipton R. B. Zonderman A. B. Resnick S. M. Kawas C. (2008). Memory impairment, executive dysfunction, and intellectual decline in preclinical Alzheimer’s disease. *J. Int. Neuropsychol. Soc.* 14 266–278. 10.1017/S1355617708080302 18282324 PMC2763488

[B18] Herting M. M. Johnson C. Mills K. L. Vijayakumar N. Dennison M. Liu C. et al. (2018). Development of subcortical volumes across adolescence in males and females: A multisample study of longitudinal changes. *Neuroimage* 172 194–205. 10.1016/j.neuroimage.2018.01.020 29353072 PMC5910239

[B19] Jacobs E. G. Weiss B. Makris N. Whitfield-Gabrieli S. Buka S. L. Klibanski A. et al. (2017). Reorganization of functional networks in verbal working memory circuitry in early midlife: The impact of sex and menopausal status. *Cereb. Cortex* 27 2857–2870. 10.1093/cercor/bhw127 27178194 PMC6059144

[B20] Kang K. Zhang P. Dumitrescu L. Mukherjee S. Lee M. L. Choi S. E. et al. (2025). The dynamics of cognitive decline toward Alzheimer’s disease progression: Results from ADSP-PHC’s harmonized cognitive composites. *Alzheimers Dement.* 21:e70335. 10.1002/alz.70335 40538025 PMC12179338

[B21] Kramer J. H. Delis D. C. Kaplan E. O’Donnell L. Prifitera A. (1997). Developmental sex differences in verbal learning. *Neuropsychology* 11 577–584. 10.1037//0894-4105.11.4.577 9345701

[B22] Larson E. B. Kukull W. A. Katzman R. L. (1992). Cognitive impairment: Dementia and Alzheimer’s disease. *Annu. Rev. Public Health* 13 431–449. 10.1146/annurev.pu.13.050192.002243 1599598

[B23] Li G. Larson E. B. Shofer J. B. Crane P. K. Gibbons L. E. McCormick W. et al. (2017). Cognitive trajectory changes over 20 years before dementia diagnosis: A large cohort study. *J. Am. Geriatr. Soc.* 65 2627–2633. 10.1111/jgs.15077 28940184 PMC5729097

[B24] Li R. Singh M. (2014). Sex differences in cognitive impairment and Alzheimer’s disease. *Front. Neuroendocrinol.* 35 385–403. 10.1016/j.yfrne.2014.01.002 24434111 PMC4087048

[B25] Liu F. Day M. Muñiz L. C. Bitran D. Arias R. Revilla-Sanchez R. et al. (2008). Activation of estrogen receptor-beta regulates hippocampal synaptic plasticity and improves memory. *Nat. Neurosci.* 11 334–343. 10.1038/nn2057 18297067

[B26] Ma L. Tan E. C. K. Bush A. I. Masters C. L. Goudey B. Jin L. et al. (2024). Elucidating the link between anxiety/depression and Alzheimer’s dementia in the Australian Imaging Biomarkers and Lifestyle (AIBL) study. *J. Epidemiol. Glob. Health* 14 1130–1141. 10.1007/s44197-024-00266-w 38896210 PMC11442410

[B27] Neufang S. Specht K. Hausmann M. Güntürkün O. Herpertz-Dahlmann B. Fink G. R. et al. (2009). Sex differences and the impact of steroid hormones on the developing human brain. *Cereb. Cortex* 19 464–473. 10.1093/cercor/bhn100 18550597

[B28] Peters F. Villeneuve S. Belleville S. (2014). Predicting progression to dementia in elderly subjects with mild cognitive impairment using both cognitive and neuroimaging predictors. *J. Alzheimers Dis.* 38 307–318. 10.3233/JAD-130842 23963293

[B29] Ratigan A. Kritz-Silverstein D. Barrett-Connor E. (2016). Sex differences in the association of physical function and cognitive function with life satisfaction in older age: The Rancho Bernardo Study. *Maturitas* 89 29–35. 10.1016/j.maturitas.2016.04.007 27180157 PMC4873962

[B30] Reitan R. M. (1958). Skills, validity of the trail making test as an indicator of organic brain damage. *Percept. Mot. Skills* 8 271–276. 10.2466/pms.1958.8.3.271

[B31] Ren H. Jing F. Zhou Y. Luo Z. Yu X. Jin W. et al. (2025). Utilizing multimodal models to forecast Alzheimer’s disease progression and clinical subtypes. *Health Inf. Sci. Syst.* 14:10. 10.1007/s13755-025-00407-w 41376794 PMC12686251

[B32] Rentz D. M. Weiss B. K. Jacobs E. G. Cherkerzian S. Klibanski A. Remington A. et al. (2017). Sex differences in episodic memory in early midlife: Impact of reproductive aging. *Menopause* 24 400–408. 10.1097/GME.0000000000000771 27824681 PMC5365356

[B33] Rodríguez-Camacho M. Martínez-Salmerón M. Andrade-Zumárraga L. Drzewiscki E. Romera-Morales D. Criado C. B. et al. (2025). Sex differences in cognitive reserve: Findings from a brief questionnaire in biologically confirmed Alzheimer’s disease. *Alzheimers Dement.* 21:e106927. 10.1002/alz70858_106927

[B34] Sando S. B. Melquist S. Cannon A. Hutton M. L. Sletvold O. Saltvedt I. et al. (2008). APOE epsilon 4 lowers age at onset and is a high risk factor for Alzheimer’s disease; a case control study from central Norway. *BMC Neurol.* 8:9. 10.1186/1471-2377-8-9 18416843 PMC2375917

[B35] Shanmugan S. Epperson C. N. (2014). Estrogen and the prefrontal cortex: Towards a new understanding of estrogen’s effects on executive functions in the menopause transition. *Hum. Brain Mapp.* 35 847–865. 10.1002/hbm.22218 23238908 PMC4104582

[B36] Small B. J. Rosnick C. B. Fratiglioni L. Bäckman L. (2004). Apolipoprotein E and cognitive performance: A meta-analysis. *Psychol. Aging* 19 592–600. 10.1037/0882-7974.19.4.592 15584785

[B37] Smith G. E. Pankratz V. S. Negash S. Machulda M. M. Petersen R. C. Boeve B. F. et al. (2007). A plateau in pre-Alzheimer memory decline: Evidence for compensatory mechanisms? *Neurology* 69 133–139. 10.1212/01.wnl.0000265594.23511.16 17620545

[B38] Stern Y. Gurland B. Tatemichi T. K. Tang M. X. Wilder D. Mayeux R. et al. (1994). Influence of education and occupation on the incidence of Alzheimer’s disease. *JAMA* 271 1004–1010. 10.1001/jama.1994.03510370056032 8139057

[B39] Stern Y. Zarahn E. Hilton H. J. Flynn J. DeLaPaz R. Rakitin B. (2003). Exploring the neural basis of cognitive reserve. *J. Clin. Exp. Neuropsychol.* 25 691–701. 10.1076/jcen.25.5.691.14573 12815506

[B40] Sundermann E. E. Biegon A. Rubin L. H. Lipton R. B. Mowrey W. Landau S. et al. (2016). Better verbal memory in women than men in MCI despite similar levels of hippocampal atrophy. *Neurology* 86 1368–1376. 10.1212/WNL.0000000000002570 26984945 PMC4831033

[B41] Todd M. Schneper L. Vasunilashorn S. M. Notterman D. Ullman M. T. Goldman N. (2018). Apolipoprotein E, cognitive function, and cognitive decline among older Taiwanese adults. *PLoS One* 13:e0206118. 10.1371/journal.pone.0206118 30339707 PMC6195295

[B42] van Hooren S. A. Valentijn A. M. Bosma H. Ponds R. W. van Boxtel M. P. Jolles J. (2007). Cognitive functioning in healthy older adults aged 64-81: A cohort study into the effects of age, sex, and education. *Neuropsychol. Dev. Cogn. B Aging Neuropsychol. Cogn.* 14 40–54. 10.1080/138255890969483 17164189

[B43] Vanderlip C. R. Stark C. E. L. (2024). APOE4 increases susceptibility to amyloid, accelerating episodic memory decline. *bioRxiv [Preprint]* 10.1101/2024.12.23.630203 39763904 PMC11703168

[B44] Wang L. Lou Y. Jiang Q. Wang H. Huang S. Xie Y. et al. (2025). Association between multimorbidity trajectories and subsequent cognitive decline: Evidence from two nationwide longitudinal cohorts. *J. Affect. Disord.* 391:119958. 10.1016/j.jad.2025.119958 40683537

[B45] Weiner M. W. Veitch D. P. Aisen P. S. Beckett L. A. Cairns N. J. Green R. C. et al. (2013). The Alzheimer’s disease neuroimaging initiative: A review of papers published since its inception. *Alzheimers Dement.* 9 e111–e194. 10.1016/j.jalz.2013.05.1769 23932184 PMC4108198

[B46] Williams O. A. An Y. Armstrong N. M. Kitner-Triolo M. Ferrucci L. Resnick S. M. (2020). Profiles of cognitive change in preclinical and prodromal Alzheimer’s disease using change-point analysis. *J. Alzheimers Dis.* 75 1169–1180. 10.3233/JAD-191268 32390623 PMC7561016

[B47] World Health Organization [WHO] (2026). *Dementia.* Geneva: World Health Organization.

[B48] Yang L. Qin G. Zhao N. Wang C. Song G. (2012). Using a generalized additive model with autoregressive terms to study the effects of daily temperature on mortality. *BMC Med. Res. Methodol.* 12:165. 10.1186/1471-2288-12-165 23110601 PMC3549928

[B49] Yuan M. Rong M. Long X. Lian S. Fang Y. (2024). Trajectories of cognitive decline in different domains prior to AD onset in persons with mild cognitive impairment. *Arch. Gerontol. Geriatr.* 122:105375. 10.1016/j.archger.2024.105375 38431989

