# Supplementary material for: Trajectories and timing of accelerated decline in specific memory domains preceding Alzheimer’s disease
Source: Front Aging Neurosci. 2026 Jun 26;18:1868433. doi: 10.3389/fnagi.2026.1868433 (PMC13352412; doi:10.3389/fnagi.2026.1868433)
Supplement: Supplementary file 1 [file Supplementary_file_1.docx]

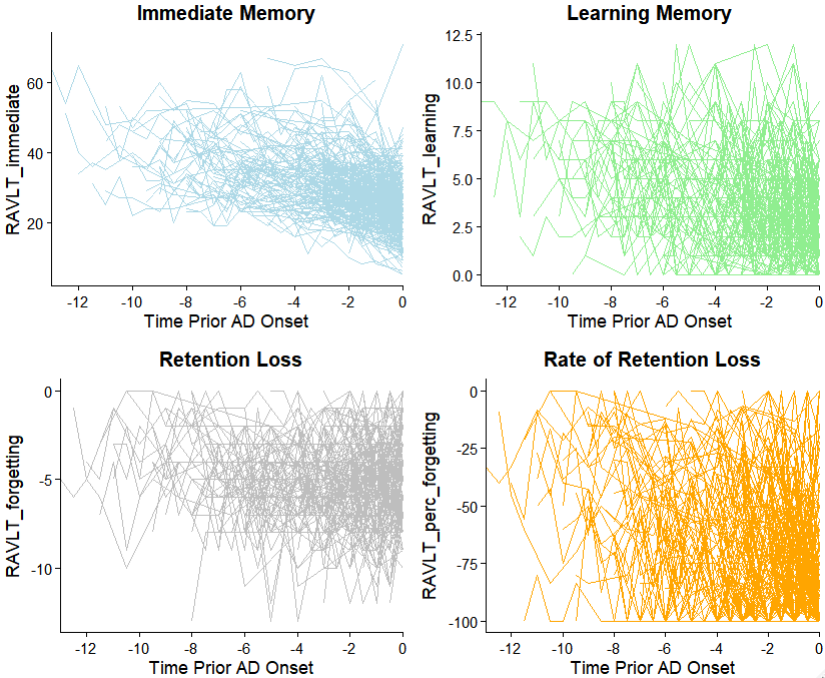


Figure S1 Participant-level raw longitudinal trajectories of four neuropsychological scores prior to AD onset


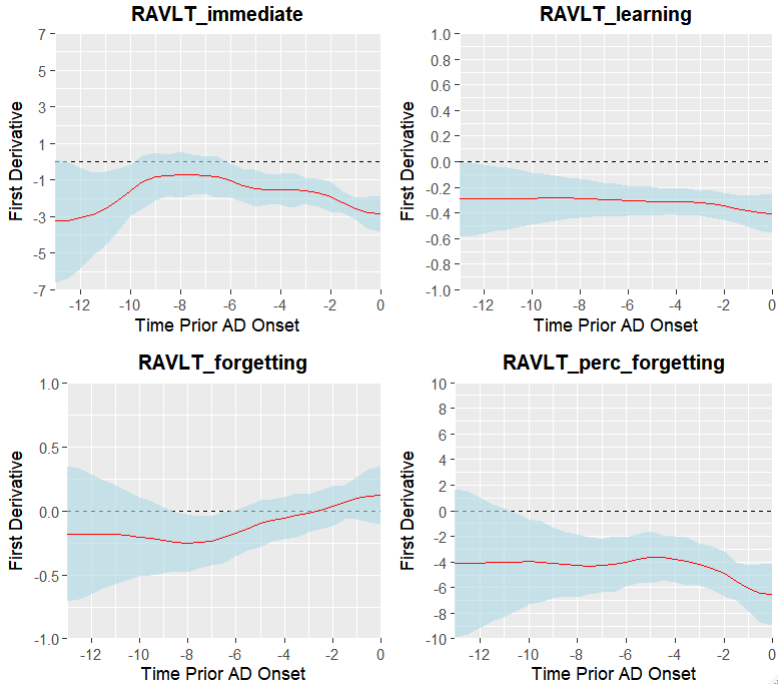


The solid red line represents the fitted first derivative curve and the blue band is the 95% confidence interval band.

Figure S2 The first-order derivative values of GAMM-fitted trajectories for four neuropsychological scores prior to AD onset

Table S1 Sensitivity Analysis of GAMM Models for Four Neuropsychological Measures with Additional Adjustment for History of Stroke and Emotional Incontinence

| **Covariates** | **RAVLT_immediate** | | **RAVLT_learning** | | **RAVLT_forgetting** | | **RAVLT_perc_forgetting** | |
| --- | --- | --- | --- | --- | --- | --- | --- | --- |
|  | ***β*** | ***p*** | ***β*** | ***p*** | ***β*** | ***p*** | ***β*** | ***p*** |
| Intercept | 29.736 | <0.001 | 2.525 | 0.045 | -5.910 | <0.001 | -83.995 | <0.001 |
| Age | -0.064 | 0.231 | -0.001 | 0.974 | 0.016 | 0.140 | 0.036 | 0.825 |
| Gender (ref = Female) |  |  |  |  |  |  |  |  |
| Male | -2.805 | <0.001 | -0.349 | 0.057 | 0.137 | 0.403 | -1.709 | 0.457 |
| Educational Year | 0.451 | <0.001 | 0.056 | 0.061 | -0.043 | 0.111 | 0.209 | 0.528 |
| Race (ref = Others^a^) |  |  |  |  |  |  |  |  |
| white | -0.292 | 0.856 | 0.294 | 0.442 | -0.080 | 0.815 | -2.088 | 0.664 |
| Marital status (ref = Others^b^) |  |  |  |  |  |  |  |  |
| Married | 0.754 | 0.423 | 0.203 | 0.364 | 0.323 | 0.108 | 4.424 | 0.116 |
| APOE-ε4 (ref =0) |  |  |  |  |  |  |  |  |
| 1 | -1.05 | 0.156 | -0.374 | 0.034 | -0.452 | 0.004 | -7.823 | 0.004 |
| 2 | -1.560 | 0.144 | -0.524 | 0.040 | -0.540 | 0.019 | -10.263 | 0.001 |
| History of Hypertension  (ref = No) |  |  |  |  |  |  |  |  |
| Yes | -0.804 | 0.253 | -0.253 | 0.131 | -0.011 | 0.940 | -1.076 | 0.609 |
| Retirement (ref = No) |  |  |  |  |  |  |  |  |
| Yes | 0.328 | 0.731 | -0.107 | 0.636 | -0.207 | 0.310 | -1.883 | 0.509 |
| History of Stroke (ref = No) |  |  |  |  |  |  |  |  |
| Yes | 3.178 | 0.159 | 0.701 | 0.197 | 0.632 | 0.198 | 13.679 | 0.044 |
| Emotional incontinence  (ref = No) |  |  |  |  |  |  |  |  |
| Yes | 3.592 | 0.221 | 1.082 | 0.120 | -0.970 | 0.121 | 1.635 | 0.852 |
| BMI | -0.044 | 0.548 | 0.002 | 0.928 | 0.030 | 0.057 | 0.368 | 0.092 |
| **Spline fit** | ***Edf*** | ***p*** | ***Edf*** | ***p*** | ***Edf*** | ***p*** | ***Edf*** | ***p*** |
| *S* (Time prior to AD) | 4.940 | <0.001 | 1.969 | <0.001 | 3.097 | <0.001 | 3.053 | <0.001 |

Note: “*S* ()” refers to the smoothing function from the generalized additive mixed models, “*Edf* ” estimated effective degrees of freedom, *p*<0.05 is significant. Data for RAVLT_immediate, RAVLT_learning, RAVLT_forgetting, and RAVLT_perc_forgetting are all longitudinal data. Data for age, education, race, marital status, history of hypertension, retirement and BMI are baseline information. a includes black people, Asians, and mixed-race individuals, b includes divorce, widowhood and unmarried. Nine had a history of stroke, and five had emotional incontinence among the 382 individuals.


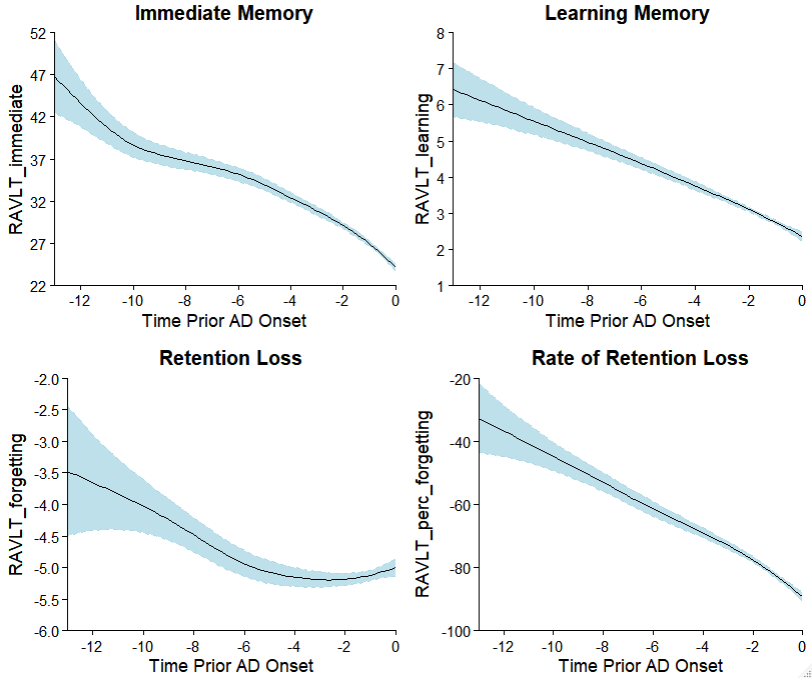


Note: The black curves represent the fitted values of GAMM. The blue band denotes the 95% confidence interval.

Figure S3 Sensitivity Analysis of Pre‑AD Onset Trajectories of Four Neuropsychological Measures from GAMM, adjusting for History of Stroke and Emotional Incontinence


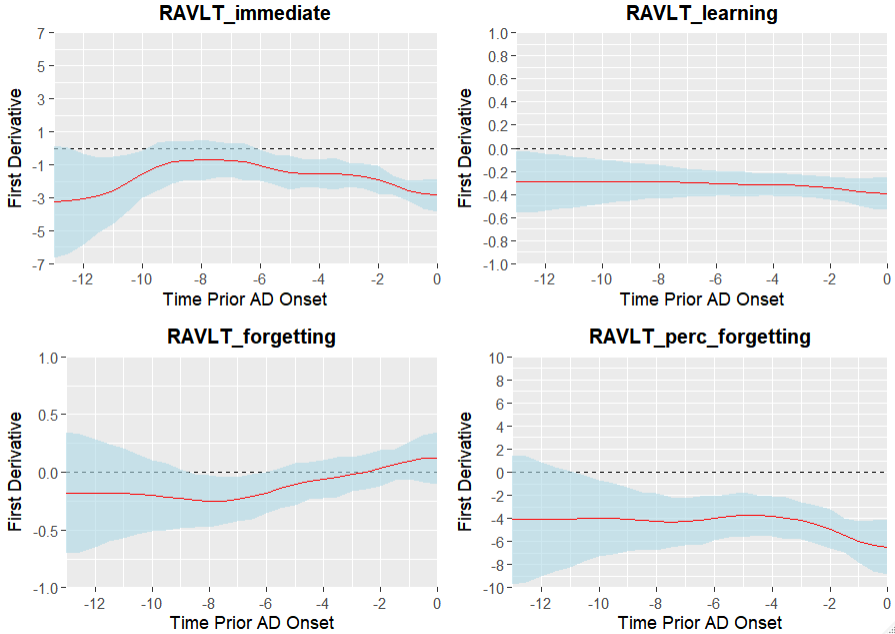


Note: The solid red line represents the first derivative curve of the fitted values of the GAMM. The blue band is the 95% confidence interval band.

Figure S4 Sensitivity Analysis of First-Order Derivative Values of GAMM-Fitted Trajectories for Four Neuropsychological Scores Prior to AD Onset, Adjusting for History of Stroke and Emotional Incontinence


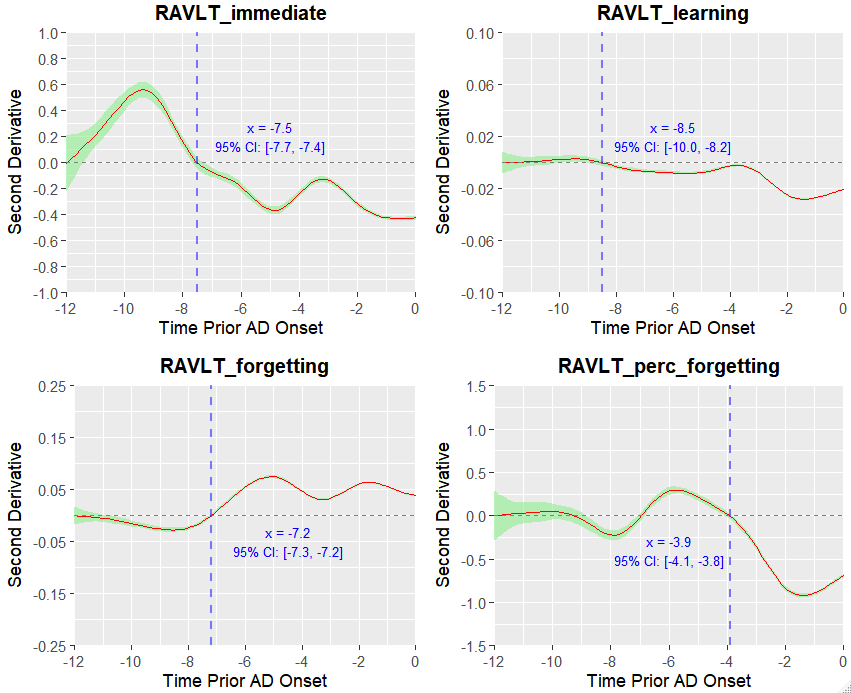


Note：The solid red line represents the fitted second derivative curve. The green band denotes the 95% confidence interval for the second derivative. The blue dashed line is the vertical line drawn at the x‑axis location of the turning point of sustained accelerated decline. The blue text indicates the 95% confidence interval for the turning point on the x-axis.

Figure S5 Sensitivity Analysis of Second-Order Derivative Values of GAMM-Fitted Trajectories for Four Neuropsychological Scores Prior to AD Onset, Adjusting for History of Stroke and Emotional Incontinence
